# Therapeutic Means of Affecting Local Inflammatory Processes

**Published:** 1884-06

**Authors:** 


### Therapeutic Means of Affecting Local Inflammatory Processes.

Dr. C. B. Nancrede concludes (*Medical News*), as a result of his investigations on this subject: 1. During the stage of dilated arteries, with increased rapidity of the current, but little danger of capillary changes with exudation need be apprehended, and here perhaps ergot, certainly arterial sedatives, do good, either directly or indirectly, without blood-letting, by reducing the size and rapidity of the current, thus allowing the veins of the irritated area time to empty themselves, even of an unaccustomed amount of blood. Thus, if vascular-pressure changes have taken place, the vessels have an opportunity to return to the normal. 2. After stasis has occurred, or while it is occurring, weakening of the heart's action and a diminished volume of the current—*e. g.*, the effect of arterial sedatives—can do nothing but harm to the inflamed area, although it may prevent extension of inflammation in the circumjacent parts, which are merely in the earlier stages of congestion. 3. The results to be sought and which are secured by local blood-letting are removal of the blood on the venous side, to that the vessels cannot only empty themselves, but a certain amount of *vis-a-fronte*, *i. e.*, aspiration—is invoked; this secondarily results not only in a temporary return to the normal on the arterial side, but an increased rapidity (and here is an important point)—lessened force of the circulation. The acceleration of rate without the weakened force of the circulation would further damage the vessels, instead of which the increased rate of the current merely serves to sweep out the accumulated red-blood cells, the cause of the excess of oxygen, and the consequent cell-migration. The vehement current also induces a rapid resorption of the effused liquor sanguinis, at once the stimulator to growth, and the food of the cells. This latter advantage is not founded on theory alone, for it is a matter of common observation that the mere amount of blood extracted produces no sensible effects on an inflamed breast, for instance, at first, but in a few hours, the skin, if carefully examined, has become wrinkled, and the organ shrunken. This effect is secondary to the loss of blood, and chiefly results from the absorption of the imflammatory exudate. 4. Arterial sedatives in the latter stages are usually inadmissible except as succedanea to blood-letting, as far as the focus of inflammation is concerned; the surrounding parts, which are merely congested, may be benefited by their exhibition. After blood-letting they act favorably, because, when the stasis has been overcome, they lessen intra-vascular pressure, and thus permit the blood-vessels to recover their normal condition. They also alleviate pain by lessening the bulk of blood in the part—*i. e.*, they relieve nerve pressure.

